# Editorial: Statistical Methods, Computing and Resources for Genome-Wide Association Studies

**DOI:** 10.3389/fgene.2021.714894

**Published:** 2021-08-05

**Authors:** Hailan Liu, Lide Han, Guolian Kang, Min Zhang, Riyan Cheng

**Affiliations:** ^1^Maize Research Institute, Sichuan Agricultural University, Chengdu, China; ^2^Vanderbilt Genetics Institute, Vanderbilt University Medical Center, Nashville, TN, United States; ^3^Department of Biostatistics, St. Jude Children's Research Hospital, Memphis, TN, United States; ^4^Department of Statistics, Purdue University, West Lafayette, IN, United States; ^5^Department of Psychiatry, University of California, San Diego, San Diego, CA, United States

**Keywords:** genome-wide association study, multi-trait analysis, nested association mapping, pleiotropy, imprinted QTL

Thanks to the recent advances in genotyping technologies, genome-wide association studies (GWAS) have been an established approach to identifying genetic variants that influence certain characteristics of economic or scientific interest in plants, animals and humans. Applications of GWAS cover a wide range of areas in genetics and have enhanced our understanding of the genetic mechanisms in diseases, physiological or behavioral traits and have generated promises in agriculture, medicine and wildlife conservation. Despite great success, GWAS remains challenged by statistical modeling and computing. This collection of twelve articles presents a variety of interesting scientific problems and novel approaches in GWAS.

Nested association mapping (NAM) is a technique for dissecting the genetic architecture of complex traits in crops. It is designed for NAM-specific populations by taking the advantages of linkage analysis and association mapping while avoiding their disadvantages. Bu et al. developed a multi-locus association mapping model for the analysis of data from multiple families in the NAM population. A notable feature of their method lies in its ability to deal with genetic heterogeneity due to subpopulations, and therefore their approach improves statistical power for quantitative trait locus (QTL) detection and accuracy of QTL effect estimation. In real data analyses, they found that their method identified most QTLs that were detected by linkage analyses of single-family datasets and was also able to disclose some new QTLs with small effects. Three of the 12 articles in this collection are concerned with multi-trait analysis. Multi-trait analysis has been of interest in GWAS due to its potential gain in statistical power and its ability for formal hypothesis testing of biological importance such as pleiotropy vs. linkage. As a common practice, multiple traits are analyzed separately and markers are scanned one at a time. Deng et al. argued that the former does not take advantage of correlations between traits and thus can be a limitation on statistical power, and the latter ignores complex interactions between genomic variants. Therefore, they proposed a genome-wide gene-based multi-trait method to overcome these limitations. Technically, they adopted kernel-based testing to evaluate the joint effect of multiple variants in a gene and proposed an omnibus test strategy to integrate the test results. They demonstrated that their method achieved excellent power with reasonable control of type I error rates. Lin et al. discussed the interpretation of the results from multi-trait analyses. They introduced a bioinformatic tool, MetaPha, which implements a meta-analysis approach by constructing multivariate analysis from univariate GWAS results and then decomposing multivariate associations into multiple ones that facilitate interpretation. They validated their method using lipid data from the Global Lipids Genetics Consortium and found that only three to five central traits of the twenty-one traits they studied were needed at the majority of the loci of their interest. Fernandes et al. focused on a biologically interesting question, linkage vs. pleiotropy, which can be tested by either multivariate or univariate approaches. Using simulation studies, they found that neither of these two approaches alone delivered a satisfactory result, and thus suggest that multivariate and univariate GWAS should be complementary rather than competing. For those interested in mapping imprinted QTLs, Zheng et al. proposed a special type of immortalized F2 population and correspondingly two methods for mapping imprinted QTL and demonstrated the merits of their proposed population and methods in mapping precision. If your research is related to Barley, you may be interested in Li et al. who studied a few traits in Qingke Barley. Other interests include heritability estimation (Xu et al. and Frouin et al.), disease studies using machine learning techniques (Fan et al. and Li et al.), and Mendelian randomization (Xu et al.).

Lastly, we would like to introduce Zhu et al. who reviewed statistical methods for identifying trait-relevant tissues and cell types. Genome-wide association studies (GWAS) have reported numerous quantitative trait loci (QTL). However, few reported QTL have been validated while the ultimate goal of GWAS is to help understand the biological mechanisms of the trait-QTL association. Some researchers have developed statistical methods to integrate genomic information with functional annotations, gene expression data, and gene network information into GWAS, and aim to identify relevant tissue and cell types. Zhu et al. extensively reviewed ten of these methods.

To conclude, this volume highlights new insights and fascinating perspectives in statistical methods, computing, and resources for GWAS. We hope the collection will stimulate more developments in this important topic as biotechnology continues to evolve.

## Author Contributions

All authors listed have made a substantial, direct and intellectual contribution to the work, and approved it for publication.

## Conflict of Interest

The authors declare that the research was conducted in the absence of any commercial or financial relationships that could be construed as a potential conflict of interest.

## Publisher's Note

All claims expressed in this article are solely those of the authors and do not necessarily represent those of their affiliated organizations, or those of the publisher, the editors and the reviewers. Any product that may be evaluated in this article, or claim that may be made by its manufacturer, is not guaranteed or endorsed by the publisher.

